# Effect of Moderate Wine Consumption on Oxidative Stress Markers in Coronary Heart Disease Patients

**DOI:** 10.3390/nu14071377

**Published:** 2022-03-25

**Authors:** Maria Choleva, Chrysa Argyrou, Maria Detopoulou, Maria-Eleni Donta, Anastasia Gerogianni, Evanggelia Moustou, Androniki Papaemmanouil, Christina Skitsa, Genovefa Kolovou, Petros Kalogeropoulos, Elizabeth Fragopoulou

**Affiliations:** 1 Department of Nutrition and Dietetics, Harokopio University, 70 Eleftheriou Venizelou Avenue Kallithea, 17671 Athens, Greece; mcholeva@hua.gr (M.C.); chrysa_argyrou@yahoo.gr (C.A.); mardeto@hua.gr (M.D.); ds215015@hua.gr (M.-E.D.); gerogianni.an@gmail.com (A.G.); elinamoustou@yahoo.gr (E.M.); ds214075@hua.gr (A.P.); ds214110@hua.gr (C.S.); 2 Cardiology Department, Onassis Cardiac Surgery Center, 17674 Athens, Greece; genovefa@kolovou.com; 3 Department of Cardiology, General Ongology Hospital, 14564 Kifisia, Greece; petros.kalo@gmail.com

**Keywords:** wine, oxidative stress, coronary heart disease, oxidized guanine species, protein carbonyls, TBARS, serum oxidation, SOD, GPx, ethanol

## Abstract

Evidence from research studies reports that wine consumption is associated with lower cardiovascular disease risk, partly through the amelioration of oxidative stress. The aim of the present study was to examine the effect of regular light to moderate wine consumption from coronary heart disease (CHD) patients compared to the effect induced by alcohol intake without the presence of wine microconstituents, on oxidation-induced macromolecular damage as well as on endogenous antioxidant enzyme activity. A randomized, single-blind, controlled, three-arm parallel intervention was carried out, in which 64 CHD patients were allocated to three intervention groups. Group A consumed no alcohol, and Group B (wine) and Group C (ethanol) consumed 27 g of alcohol/day for 8 weeks. Blood and urine samples were collected at baseline and at 4 and 8 weeks. Urine oxidized guanine species levels, protein carbonyls, thiobarbituric acid substances (TBARS) levels, as well as superoxide dismutase (SOD) and glutathione peroxidase (GPx) activities, were measured. Oxidized guanine species and protein carbonyl levels were significantly increased in the ethanol group during the intervention and were significantly decreased in the wine group. These results support the idea that wine’s bioactive compounds may exert antioxidant actions that counteract the macromolecular oxidative damage induced by alcohol in CHD patients.

## 1. Introduction

Cardiovascular disease (CVD) is the most prevalent global cause of death, leading to 17.3 million deaths annually [1], and atherosclerosis is their underlying mechanism. Oxidative stress, including the oxidative modification of low-density lipoprotein (LDL) cholesterol, is an important step in the initiation of the plaque formation [2]. Platelet Activating Factor (PAF) and oxidized phospholipids are produced during the oxidation of LDL and act as crucial inflammatory mediators, while PAF also possesses potent platelet aggregatory action [3]. The excess production of reactive oxygen species (ROS) has been associated with cardiovascular disease, which is reflected in the modified macromolecules structure and functionality, as DNA, proteins, and lipids are crucially affected by oxidative processes [4]. Antioxidant and pro-oxidant enzymes play an important role in the maintenance of ROS in physiological levels and the over-function of pro-oxidant enzymes versus antioxidant ones, and they also promote oxidative stress and prolongation of disease.

The beneficial properties of wine consumption were firstly established in 1979 when an inverse association between red wine consumption and CVD mortality was reported [5]. Since then, scientific research has provided abundant evidence regarding the association between moderate wine consumption and stroke incidence, as well as CVD mortality [6,7,8]. The health effects of drinking are highly associated with the quantity and pattern of ethanol consumption. Epidemiological studies consistently find that light and moderate drinking has been linked to favorable outcomes in primary prevention [9] and in subjects with established CVD [10,11]. The beneficial effects of wine consumption are attributed not only to its alcohol content but also and mainly to its bioactive compounds that exert potent antioxidant, anti-inflammatory, and anti-thrombotic effects [12]. Previous studies of our research group have reported that wine extracts, as well as phenolic compounds that are present in wine, exert antioxidant actions, inhibit the in vitro PAF-induced platelet aggregation, and reduce the activity of PAF biosynthetic enzymes in U937-monocytes [13,14,15,16,17,18,19,20]. 

Apart from epidemiological data, both acute and long-term effects of wine consumption have been investigated in randomized controlled clinical trials concerning metabolic and physio-pathological systems related to CVD [21]. Specifically, on the antioxidant effects of wine consumption, many clinical trials provide evidence for potential protective effects of moderate consumption in healthy volunteers, despite the extensive differences in the methods and biomarkers used among the studies [7,22,23,24,25,26,27]. Despite the promising data, these studies were conducted on healthy volunteers, and their results cannot be interpreted on subjects with CVD. Fewer studies do investigate such patients but do not include a control group consuming an equal amount of alcohol in order to distinguish the biological effects of microconstituents present in wine from those of alcohol [28,29]. In our previous publication, the light to moderate consumption of wine for 8 weeks revealed an attenuation of the ethanol consumption effect on cytokine secretion at basal conditions from CVD patients’ peripheral blood mononuclear cells [30]. 

Therefore, the aim of the present study is to examine the effect of regular light to moderate wine consumption from CVD patients, compared to the effect induced by alcohol intake without the presence of wine microconstituents, on oxidation-induced macromolecular damage as well as on endogenous antioxidant enzymes’ activity.

## 2. Materials and Methods

### 2.1. Reagents and Chemicals

All reagents and chemicals used were of analytical grade. All reagents and solvents were supplied from Sigma (St. Louis, MO, USA).

### 2.2. Participants of the Study

The participants of the study were male coronary heart disease (CHD) patients with at least 6-month stable medication and weekly estimated alcohol consumption between 10 and 28 g. Diagnosis of CHD was established by angiography or the presence of one of the subsequent criteria: positive stress test, positive myocardial perfusion scintigraphy with thallium, positive triplex heart ultrasound with dobutamine, or hospitalization due to myocardial infarction or stroke. Patients with diabetes, renal or hepatic diseases, acute respiratory infections, cold, flu, or medical history of other inflammatory diseases were excluded from the study. Seventy-one patients were initially recruited under the supervision of their cardiologists, from hospitals in Athens, Greece, and 64 of them were randomly assigned to one of the three study groups after meeting the inclusion criteria.

### 2.3. Trial Design

In order to evaluate if light to moderate wine consumption from CHD patients may affect oxidative stress compared to the consumption of an equal amount of alcohol, a randomized, single-blind, controlled, 3-arm parallel intervention study was carried out. The participants were asked to abstain from alcohol 15 days prior to the intervention, and then they were randomly assigned to one of the three groups. The randomization was carried out by someone impartial towards the study staff member using computer-generated random numbers. The volunteers were asked to maintain their usual dietary and physical activity habits as well as their prescribed medication. In Group A (control group), the subjects abstained from alcohol; in Group B (wine group), they consumed 200 mL of red wine (variety Cabernet Sauvignon 13.5% *v*/*v* alcohol); and in Group C (ethanol group), the subjects consumed 71 mL of tsipouro (a Greek alcoholic drink produced by distillation) with 38% *v*/*v* alcohol per day. The amount of alcohol in the last 2 groups was equal to 27 g of ethanol per day, consumed along with lunch or dinner. The study was approved by the Bioethics Committee of Harokopio University and was carried out in accordance to the Declaration of Helsinki (1989). The study took place at the Department of Nutrition and Dietetics of Harokopio University in Athens, Greece, after the participants had given written consent. The intervention was also registered in ClinicalTrials.gov (NCT04438122). The intervention period for each volunteer was 8 weeks, and blood samples were collected at the beginning (0 week), the middle (4 week), and the end (8 week) of each intervention. Smoking habits were recorded through a standard questionnaire. A total of 57 subjects completed the study: 20 in Group A (2 dropouts), 21 in Group B (1 dropout) and 16 in Group C (4 dropouts). 

### 2.4. Dietary and Physical Assessment of the Volunteers

In order to assess the participants’ compliance with the study, dietitians collected three 24 h recalls at baseline, three at the 4th and three at the 8th week of the intervention. These data were analyzed for their energy, macro- and micronutrient content using the Nutritionist Pro, version 2.2, software (Axxya Systems-Nutritionist Pro, Stafford, TX, USA). A validated food frequency questionnaire [31] was used in order to assess the habitual dietary intake and alcohol consumption. These data were used to estimate the level of adherence to the Mediterranean diet through the MedDietScore [32]. Physical activity of the subjects was assessed with the use of a validated questionnaire (IPAQ-short version) and was expressed in metabolic equivalent minutes per week (MET-min-week) [33]. 

### 2.5. Anthropometric Measurements and Blood Sample Collection

Anthropometric measurements were made on each visit of every subject. Participants’ weight was measured to the nearest 0.1 kg using a digital scale and height to the nearest 0.1 cm using a stadiometer (in light clothing and without shoes). Waist circumference was measured to the nearest 0.1 cm between the superior iliac crest and the lower rib margin in the mid axillary line. Resting arterial blood pressure was measured three times in the right arm with the subject in sitting position. Blood samples collection was held after 12 h fasting. Complete blood count was determined in ethylene-diamine-tetra acetic acid (EDTA) anticoagulated whole blood with a Mindray BC-3000 hematology analyzer (Mindray, Shenzhen, China). Serum and plasma were isolated and frozen at −80 °C. Urine samples were also collected and frozen at −80 °C.

### 2.6. Basic Biochemical Measurements

Glucose levels, total cholesterol (TC), triglycerides (TG), and high-density lipoprotein (HDL) cholesterol were measured in serum samples using an enzymatic colorimetric assay (COBAS^®^ 8000 analyzer, Roche Diagnostics International AG, Rotkreuz, Switzerland). LDL cholesterol levels were calculated using the Friedewald formula. Insulin levels were determined by the chemiluminescence method (E170 modular analyzer, Roche). The levels of liver enzymes, namely gamma glutamyltransferase (γ-GT), serum glutamic oxaloacetic transaminase/aspartate aminotransferase (SGOT/AST), and serum glutamic pyruvic transaminase/alanine aminotransferase (SGPT/ALT), were measured by the enzymatic colorimetric method (COBAS^®^ 8000 analyzer, Roche).

### 2.7. Protein Carbonyls

Serum samples were initially analyzed for their protein content using the Bradford method [34]. A 0.1 mL measure of 2,4-dinitrophenylhydrazine (DNPH) 10 mM was added in serum containing 1 mg protein. Samples were incubated for 10 min at room temperature, and then 0.03 mL trichloroacetic acid (TCA) solution 100% was added. After 5 min incubation in ice, the samples were centrifuged (13,000× *g*, 2 min) in order to remove the supernatant. A 0.5 mL measure of cold acetone was then added to the sediments, and after a 30 s sonication process, they were incubated in −20 °C for 5 min. Samples were centrifuged (13,000× *g*, 10 min) in order to remove the supernatant and the sediments were dissolved in 0.2 mL guanidine hydrochloride 6 M. A 0.1 mL measure of each sample was transferred into a 96-well plate, and absorbance was measured at 375 nm. In order to estimate the remaining protein content in the samples, absorbance was read at 280 nm. The analysis was conducted with the use of a microplate spectrophotometer (BioTek PowerWave XS2, Agilent Technologies, Highland Park, TX, USA). Results were reported as nmol protein carbonyls per μg of protein. 

### 2.8. Thiobarbituric Acid Reactive Substances (TBARS)

TBARS levels were measured in serum using a modified colorimetric method [35]. Briefly, 0.1 mL of serum was added to 0.2 mL of phosphoric acid 0.2 Μ, 0.025 mL of butylated hydroxyl toluene (BHT) 5 mΜ, and 0.025 mL thiobarbituric acid 0.11 M. The mixture was incubated for 60 min at 90 °C and cooled, and after the addition of 0.5 mL butanol, it was centrifuged (12,000× *g*, 10 min). The butanolic phase was collected, centrifuged (12,000× *g*, 10 min), and transferred in a 96-well plate and the absorbance was measured at 532 nm. The analysis was conducted with the use of a microplate spectrophotometer (BioTek PowerWave XS2). TBARS concentration was calculated using 1,1,3,3-tetramethoxypropane as a standard. Results are expressed as μM.

### 2.9. DNA/RNA Oxidation

Urine oxidized guanine species (8-hydroxy-2′-deoxyguanosine, 8-hydroxyguanosine and 8-hydroxyguanine) were determined by means of a competitive ELISA, using a commercially available kit (Cayman Chemicals, Ann Arbor, MI, USA). The intra- and interassay variation coefficients were 15.5% and 5.5%. Urinary creatinine (Cr) levels (mg/mL) were measured in urine samples using the Jaffé method (Biosis Creatine Jaffé-Kinetics, Biotechnological Applications Ltd., Athens, Greece) and were used as an index of standardization.

### 2.10. Superoxide Dismutase (SOD) Activity

Total SOD activity was measured in leukocyte-rich plasma (LRP) according to the method of McCord et al. [36]. The sample volume was adjusted until the inhibition was achieved between 40 and 60%, representing the linear part of the inhibition curve. Units were calculated based on the formula SOD Units = %inhibition/(100%inhibition), and results are expressed as units per mg of protein.

### 2.11. Glutathione Peroxidase (GPx) Activity

The GPx activity was measured in serum and in LRP by continuous monitoring of the regeneration of reduced glutathione (GSH) from oxidized glutathione (GSSG) upon the action of glutathione reductase (GR) and NADPH [37]. The analysis was conducted with the use of a microplate spectrophotometer (BioTek PowerWave XS2). Results were expressed as units of GPx per mL of serum (U/mL) and as units of GPx per mg of protein (U/mg).

### 2.12. Statistics

The Kolmogorov–Smirnov test was used in order to assess normal distribution. Normally distributed variables are expressed as mean ± standard deviation, while skewed variables are expressed as median (25th–75th). Variables with normal distribution at base-line were analyzed with the one-way analysis of variance (ANOVA) test, whereas skewed variables were analyzed with the Kruskal–Wallis test. For normally distributed variables, comparisons of the response curves of the three groups were performed using the repeated-measures ANOVA (RMANOVA) to test the time effect (P_time_ accounts for the differences in the same group, among the three time points of the intervention 0, 4, and 8 weeks), the trial effect (P_trial_ is referred to the differences among the three intervention groups), and the time × trial interaction (P_time*trial_, accounts for differences among the three intervention groups over time). Multiple pairwise comparisons were performed using the Bonferroni correction. The differences among intervention groups at 4 and 8 weeks for skewed variables were revealed using the Kruskal–Wallis test in order to reveal a potential trial effect (P_trial_), while the changes over time within each intervention group were analyzed using the Friedman’s 2-way ANOVA by ranks (P_time_). In order to assess the differences within each group compared to baseline values, the paired *t*-test and Wilcoxon test for paired samples were performed. Analyses were initially performed in terms of the intention to treat (ITT) the population, for which missing values were predicted using estimating-equation methods by fitting a statistical model to the observed data [38]. A secondary complete-case analysis was also performed in the per-protocol (PP) population. The SPSS statistical software package, version 18, was used for all the statistical calculations. In the case of normally distributed variables, that RMANOVA was performed, the *p* represents the *p* trend, but in all other statistic tests that were performed (ANOVA, Kruskal–Wallis, Friedman), *p* represents the *p* value. All the reported *p*-values were compared with a significance level of 5%. 

## 3. Results

**Figure 1:**
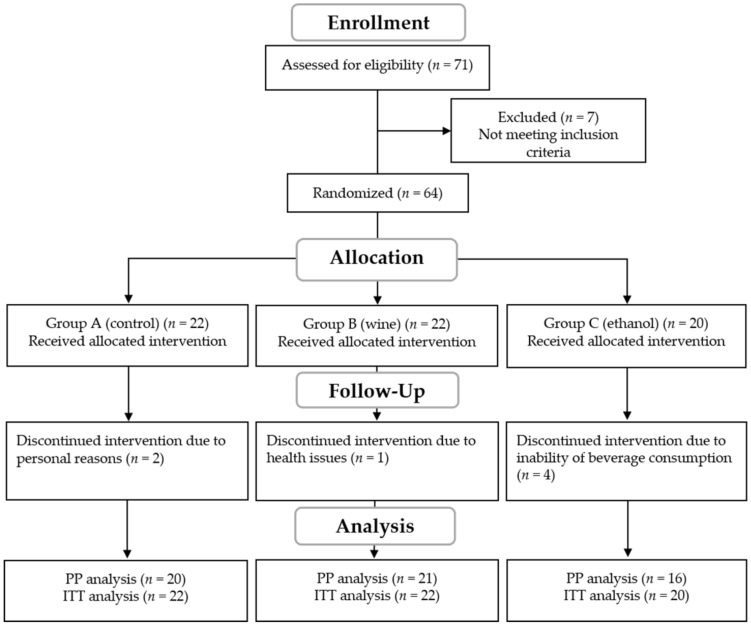
Flowchart of the intervention. PP; per protocol, ITT; intend to treat.

The trial flowchart of the study including the ITT analysis is presented in Figure 1. A total number of 57 participants completed the study, including 20 in Group A, 21 in Group B, and 16 in Group C, aged between 36 and 81 years old. A PP analysis of the results was performed for the 57 participants who completed the study, as well as an ITT for the 64 participants who were initially allocated into the intervention groups.

### 3.1. Basic Biochemical Characteristics of the Participants at Baseline

The participants’ basic biochemical data at baseline did not differ between the three intervention groups (Table 1). The participants’ values were within the normal range except for BMI, indicating an overweight study sample.

### 3.2. Oxidative Stress Biomarkers’ Levels of the Participants at Baseline

No difference was observed in baseline values of oxidative stress biomarkers between the three intervention groups (Table 2). However, a trend for higher protein carbonyls levels was observed in the participants of the wine consumption group (*p* = 0.088).

### 3.3. Basic Biochemical Characteristics of the Participants during Intervention

In Table 3, the % change of baseline levels for basic biochemical markers are presented. No differences were observed between the basic biochemical values except for insulin, which was reduced in the ethanol group at the 8th week of the intervention compared to baseline values (P_time 0–8_ = 0.043) but not compared to the other groups.

### 3.4. DNA/RNA Oxidation

The DNA/RNA oxidation products were measured in urine samples. A significant modulation in oxidized guanine species levels was observed for participants in the wine group (P_time_ = 0.012). Specifically, a decrease by 24.4% at 4 weeks (P_time 0–4_ = 0.008) and by 15% at 8 weeks (P_time 0–8_ = 0.042) compared to baseline values were observed. On the contrary, oxidized guanine species levels in the ethanol group were significantly (P_time_ = 0.002) increased, and they were especially increased at 8 weeks by 31.1% (P_time 0–8_ < 0.000) compared to baseline values. The reduction observed in the wine group was significant compared to ethanol group at 4 weeks (decrease by 33%, P_trial B-C_ = 0.003) and at 8 weeks (decrease by 46.1% P_trial B-C_ < 0.000), whereas there were no differences compared to the control group. The increase in the ethanol group at 8 weeks was also significant compared to the control group (increase by 40.5%, P_trial A-C_ < 0.000) (Table 4). 

### 3.5. Protein Carbonyls

Protein oxidation was estimated by measuring protein carbonyls in serum. Protein carbonyls levels in the wine group significantly decreased (P_time_ = 0.001). Specifically, their levels decreased at 4 weeks by 10.5% (P_time 0–4_ < 0.000) and at 8 weeks by 15.6% (P_time 0–8_ = 0.001) compared to baseline values. Additionally, an increase that was observed at 8 weeks in the ethanol group by 18.1% was significant compared to baseline values (P_time 0–8_ = 0.044). The reductions in the wine group were also significant compared to the other interventions. Specifically, at 4 weeks, they were decreased by 7.9% compared to control (P_trial A-B_ = 0.009) and by 21.7% compared to the ethanol group (P_trial B-C_ < 0.000), and at 8 weeks, they were reduced by 33.7 % compared to the ethanol group (P_trial B-C_ = 0.002) (Table 4). 

### 3.6. Lipid Oxidation

For lipid peroxidation, TBARS levels in serum were measured. No significant overall changes over time or among groups were observed concerning the TBARS levels (P_time_ = 0.197, P_trial_ = 0.775, P_time*trial_ = 0.898) (Table 4).

### 3.7. Antioxidant Enzymes’ Activities

In order to obtain a more comprehensive picture of the volunteers’ redox status during the intervention the activity of the main antioxidant enzymes, namely GPx in serum, SOD, and GPx in LRP, was measured. Although serum GPx activity was not changed overall over time in any intervention group, a slight but significant decrease by 2.4% in the wine group at 4 weeks was revealed in paired comparisons compared to baseline values (P_time 0–4_ = 0.039). Additionally, the same pattern was observed for LRP GPx activity in the wine group that was significantly decreased by 15.7% at 8 weeks compared to 4 weeks of the intervention (P_time 4–8_ = 0.024). However, their activities were not significantly different among the three groups (Table 4). 

### 3.8. Per-Protocol Analysis

The per-protocol analysis revealed a similar trend but less potent results. Concerning oxidized guanine species, they were not significantly increased in the ethanol group at 8 weeks. Protein carbonyls in the wine group at 4 weeks were decreased only compared to the ethanol group and not to the control group, whereas no differences were observed at 8 weeks among groups or compared to baseline. The time effect of the decreased insulin levels in the ethanol group was not evident in this analysis, whereas a reduction in SGPT levels was revealed in the same group, probably due to the small sample size. 

## 4. Discussion

In this study, we observed that patients who consumed moderate amounts of wine for 8 weeks had favorable effects concerning DNA/RNA and protein oxidative damage. To the best of our knowledge, the current study is the first to evaluate the effect of wine consumption based on a large number of oxidative stress biomarkers in CVD patients compared to similar populations that abstained from alcohol or consumed another alcoholic beverage for the same period.

Oxidative stress is considered to be a crucial causative factor in the onset of CVD, as plenty of interlinked cellular metabolic pathways that contribute to the development of disease are strictly determined by ROS. Total antioxidant capacity was significantly lower in 100 patients with ischemic stroke [39] and 77 CHD patients [40] compared to the healthy control group. The oxidative stress induced by increased function of enzymes responsible for the production of ROS, along with a simultaneous decrease in antioxidant pathways, promotes the oxidative modification of DNA/RNA, lipids, and proteins [41]. Several clinical studies have shown that oxidized products of macromolecules are elevated in subjects with CVD [42,43,44,45,46]. 

Epidemiological data support moderate alcohol consumption in patients with established CVD towards secondary prevention. A meta-analysis of 8 prospective studies including 16,351 diagnosed patients revealed a J-shaped curve association, with maximal observed risk reduction seen at alcohol intakes of about 26 g/day (amount equal to two drinks) [10]. Another epidemiological study of patients with myocardial infarction has also reported the same J-shaped relationship between alcohol intake and CVD events or mortality [11]. Apart from epidemiological studies [8], there is evidence reported in clinical trials that light to moderate wine consumption exerts beneficial effects in healthy volunteers through amelioration of oxidative stress [7,22,23,24,25,26,27], but few data exist that apply to populations with CHD. In general, data obtained from controlled clinical trials reveal that wine consumption may be beneficial towards antioxidant status in CHD patients [28,29]. Nevertheless, it is unclear whether the reported effects were attributed to alcohol or wine microconstituents. 

In the present study, we included two separate control groups in order to investigate the effect of wine compounds (wine group), ethanol (ethanol group, which consumed the traditional Greek alcoholic beverage namely tsipouro), and abstinence (control group, which abstained from alcohol) in CHD patients’ oxidative stress biomarkers. The alcoholic beverages were well-tolerated, and no significant effects were observed on fasting glucose levels, lipid profile, or the activity of liver enzymes. To our surprise, fasting insulin levels were significantly decreased by 6.6% compared to baseline levels after 8 weeks of tsipouro consumption, but this reduction was not significant compared to the other intervention groups. A recent meta-analysis has also reported a reduction in fasting insulin levels by moderate long-term alcohol consumption, but this conclusion was drawn by clinical trials that included both wine and other alcoholic spirits as well as healthy volunteers and pathological populations [47].

Studies have demonstrated that DNA oxidation products facilitate the development and the progression of atherosclerosis [43]. Significantly higher 8-hydroxy-2′-deoxyguanosine (8-OHdG) levels or other oxidized guanine species were observed in CHD patients compared to healthy control subjects [43,44,45]. Overall DNA damage was elevated even in premature CHD, along with CHD patients compared to age-matched control subjects [46]. Similar results concerning 8-OHdG levels were revealed for myocardial infarction patients compared to healthy controls [48]. It has been shown that 8-OHdG levels may decrease after wine consumption [7]. The only study in CHD patients by Guarda et al. [28] found that participants who consumed 250 mL of red wine for 12 months decreased their 8-OHdG levels compared to the CHD patients who abstained from alcohol. In the present study, multiple oxidized guanine species in urine, including 8-hydroxyguanosine (8-OHG), 8-OHdG, and 8-hydroxyguanine, were measured. The results clearly reveal the harmful effect of ethanol and the beneficial effect of moderate wine consumption on DNA/RNA oxidation. After 8 weeks of alcohol consumption in the form of tsipouro, oxidized guanine species levels were elevated compared to baseline and the other groups. On the contrary, after 4 and 8 weeks of wine consumption, oxidized guanine species were significantly decreased compared to baseline levels and compared to the ethanol group. In this regard, although studies cannot be directly compared due to different design, alcohol consumption tends to increase DNA damage in contrast to wine consumption [49]. Based on the above data, it is reasonable to assume that wine’s protective effect on DNA/RNA oxidation is mainly attributed to its microconstituents. 

Protein carbonylation is likely to occur through direct oxidation of lysine, arginine, proline, and threonine residues, contributing to oxidative stress intensification as well as endothelial dysfunction. Several mechanisms of abdominal aortic aneurysms, including hypertension and increased activity of metalloproteinases, may be linked to oxidative procedures [50]. Additionally, protein oxidation adducts inhibit 26S proteasomal activity, resulting in intracellular accumulation of damaged proteins. Protein carbamylation contributes to a decrease in cardiac and kidney functions and increased myocardial apoptosis after ischemia/reperfusion shown in humans [51]. Older participants have been reported to demonstrate increased levels of carbamylated proteins compared to younger subjects in a research study [52]. Premature CHD and CHD patients had higher protein carbonyl levels compared to age-matched control subjects [46]. Marfella et al. reported that diabetic patients with a myocardial infarction who consumed a moderate amount of red wine for 12 months decreased their nitrotyrosine levels (another protein oxidation marker) compared to patients who abstained from alcohol [29]. As far as we know, there are no data concerning the effect of wine consumption on protein carbonyl levels. In the present study, after 8 weeks of ethanol consumption, protein carbonyl levels were elevated compared to their baseline values. On the contrary, wine consumption led to the reduction of protein carbonyls levels at 4 and 8 weeks compared to baseline values. This reduction was also significant compared to control and ethanol group at 4 weeks, while at 8 weeks, it was significant compared only to ethanol group. 

Malondialdehyde (MDA), produced by lipid peroxidation, is able to react with the ApoB100 protein, a constituent of the LDL molecular complex [53], as well as other proteins and nucleic acids, leading to additional oxidative damage of cell components [39]. Significantly increased MDA levels have been found in patients with hypertension, cerebrovascular diseases or CHD [41]. Many studies investigating CHD patients have reported elevated MDA levels compared to healthy control subjects [40,43,54,55,56]. Similar higher MDA levels compared to control group were also reported for hypertensive elders [57]. Surprisingly, MDA levels were higher in premature CHD patients compared to controls and not in CHD patients, while its levels did not differ in CHD patients compared to the control group [46]. In our study, TBARS levels did not differentiate after the intervention, in contrast to another study where MDA levels were reduced after 2 weeks of wine consumption in healthy young and older adults [23]. 

Concerning antioxidant protection, enzymes such as SOD, GPx or catalase are important factors for the reduction of oxidative damage, as well as for the investigation of redox signaling pathways. It has been suggested that in the early stages of CHD, an upregulation of the enzymatic antioxidant defense occurs in response to the increased load of ROS in order to prevent lipid oxidation and vascular damage, whereas in the later stages, the chronically elevated levels of ROS lead to decreased enzymatic activity [42,58]. Plenty of research studies investigating CHD patients have reported lower SOD activity levels compared to healthy control subjects [40,54,55,56,59,60]. Fewer ones report higher SOD activity levels [58,61] or no differences [43,46] compared to the control group. Conflicting results exist concerning GPx activity in CHD patients, as authors report increased [43], decreased [54], or similar [46] activity levels compared to healthy control subjects. In the present study, only GPx activity was slightly decreased at certain time points during the wine intervention, in contrast to another study, in which GPx activity was increased and SOD activity was decreased after moderate white wine consumption for one month by healthy volunteers [24]. SOD activity was also decreased in a cross-over study after long-term moderate red wine consumption compared to gin consumption by healthy volunteers [26]. 

According to the results of the present study, we can hypothesize that wine’s bioactive compounds are responsible for the observed reduction in DNA and protein oxidation-derived products. It is worth noticing that in the same population, the light to moderate wine consumption for 8 weeks revealed an attenuation of the effect of ethanol consumption on cytokine secretion at basal conditions from the patients’ peripheral blood mononuclear cells [30]. Phenolic compounds present in wine are capable of scavenging free radicals (such as superoxide anion and peroxynitrite) as well as chelate transition metals involved in free radical formation [18]. Additionally, polyphenols can interact with membrane receptors or enzymes and initiate a series of redox-dependent reactions, resulting in the modification of the redox status of the cells [62]. Clinical interventions that investigated wine consumption revealed that the observed antioxidant effects were related to the elevation of the concentration of polyphenols in plasma [23,26,27]. Apart from the rich polyphenolic content, wines are abundant in a variety of other water- or lipid-soluble micro-constituents, including phospho- and glyco-lipids, with proven biological actions in vitro [18,19]. It should be noted, however, that based on the study’s design, the observed biological activity cannot be attributed to a specific component or group of components. Additionally, the observed effect may be due to either the initial compounds found in wine or due to their metabolites. It is known that after consumption, polyphenols are subjected to methylation, sulfation, or glucuronidation in the liver, and then their metabolites are released in circulation [62]. In addition, the ethanol content may have facilitated the absorption and metabolism of wine’s bioactive compounds since plasma antioxidant capacity increased by 17% in healthy men, 1 h after red wine compared to de-alcoholized red wine consumption, despite the similar antioxidant capacity of both beverages [25].

## 5. Conclusions

In conclusion, light to moderate daily consumption of wine for 8 weeks is well-tolerated and does not have any adverse effects on the basic biochemical markers in cardiovascular disease patients or any favorable effect on lipid peroxidation and endogenous antioxidant enzymes’ activity. However, wine’s bioactive compounds seem to attenuate the effect of ethanol on DNA and protein oxidation. Our data support the claim that the protective effect of moderate wine consumption can be partially attributed to its micro-constituents’ antioxidant effect, and appropriately designed clinical studies need to be conducted in order to confirm these findings.

## Figures and Tables

**Table 1:** Basic biochemical and anthropometric characteristics before intervention (0 weeks).

	Group A Control	Group B Wine	Group C Ethanol	*p* ^†^
Age (years)	63.1 ± 11.1	61.2 ± 11.6	61.7 ± 12.9	0.853
BMI (kg/m^2^)	29.3 (27.5–36.8)	28.8 (26.4–30.7)	29.1 (25.0–32.4)	0.412
Systolic blood pressure (mmHg)	131.9 ± 17.0	136.0 ± 18.9	141.1 ± 10.4	0.260
Diastolic blood pressure (mmHg)	72.2 ± 8.6	75.4 ± 14.0	78.6 ± 9.4	0.260
Glucose (mg/dL)	107.0 (94.3–122.0)	108.5 (97.0–151.3)	109.5 (97.3–122.8)	0.728
Triglycerides (mg/dL)	112.0 (77.8–170.0)	109.5 (93.5–159.5)	105.5 (86.5–156.3)	0.983
Total Cholesterol (mg/dL)	158.5 ± 37.3	170.0 ± 31.7	166.1 ± 33.7	0.538
HDL-c (mg/dL)	43.5 ± 9.8	48.1 ± 14.8	45.1 ± 11.5	0.454
LDL-c (mg/dL)	89.8 (61.5–106.8)	89.7 (74.4–109.8)	84.1 (77.1–106.8)	0.778
Uric acid (mg/dL)	6.3 (4.9–7.6)	5.9 (5.3–6.2)	6.1 (5.4–7.5)	0.450
Insulin (μΙU/mL)	11.0 (7.3–18.6)	9.7 (7.0–13.9)	8.2 (6.5–11.4)	0.458
HOMA IR	2.7 (1.9–4.9)	2.7 (2.4–3.7)	2.2 (1.9–3.3)	0.543
SGOT-AST (IU/L)	19.0 (17.8–23.3)	19.5 (16.8–22.0)	19.5 (16.3–24.8)	0.960
SGPT-ALT (IU/L)	19.5 (14.0–29.8)	19.0 (11.8–26.3)	20.0 (13.5–23.5)	0.628
γGT (IU/L)	23.0 (16.0–34.3)	23.0 (16.8–33.0)	21.5 (18.3–40.8)	0.719

Data are presented as means ± SD for normally distributed variables or as median (25th–75th quartiles) for skewed variables. One-way ANOVA or Kruskal–Wallis tests were used for the comparisons, respectively. BMI; body mass index, HDL; high-density lipoprotein, LDL; low-density lipoprotein, HOMA IR; homeostatic model assessment for insulin resistance, γ-GT; gamma glutamyltransferase, SGOT/AST; serum glutamic oxaloacetic transaminase/aspartate aminotransferase, SGPT/ALT; serum glutamic pyruvic transaminase/alanine aminotransferase. ^†^ *p* value from ANOVA or Kruskal–Wallis.^.^

**Table 2:** Oxidative stress biomarkers’ levels before intervention (0 weeks).

	Group A Control	Group B Wine	Group C Ethanol	*p* ^†^
Oxidized guanine species (μg/mmol creatinine)	16.6 (12.6–22.8)	22.4 (11.1–27.1)	11.7 (8.7–19.8)	0.153
TBARS (μM)	1.44 ± 0.47	1.48 ± 0.43	1.51 ± 0.34	0.831
Protein carbonyls (nmol/mg protein)	0.83 (0.69–0.97)	0.89 (0.79–1.21)	0.83 (0.73–0.97)	0.088
Serum GPx activity (U/mL)	0.096 (0.073–0.116)	0.108 (0.094–0.116)	0.089 (0.073–0.114)	0.133
LRP GPx activity (U/mg)	0.0029 (0.0021–0.0107)	0.0066 (0.0025–0.0106)	0.0030 (0.0022–0.0039)	0.168
SOD activity (U/mg)	2.3 (1.1–3.2)	2.3 (1.3–3.2)	2.5 (1.6–4.1)	0.596

Data are presented as means ± SD for normally distributed variables or as median (25th–75th quartiles) for skewed variables. One-way ANOVA or Kruskal–Wallis tests were used for the comparisons, respectively. TBARS; thiobarbituric acid substances, GPx; glutathione peroxidase, LRP; leukocyte-rich plasma, SOD; superoxide dismutase. ^†^ *p* value from ANOVA or Kruskal–Wallis.

**Table 3:** % Change of baseline values in basic biochemical and anthropometric characteristics.

	Group	% Change 4 Weeks	% Change 8 Weeks	P_time_^†^	P_trial_^†^ 4 Weeks	P_trial_^†^ 8 Weeks
BMI (%)	Group A Control	100.4 (99.6–101.0)	100.0 (99.6–101.3)	0.154	0.872	0.924
	Group B Wine	99.8 (99.2–101.3)	100.0 (99.1–100.9)	0.861		
	Group C Ethanol	100.7 (99.2–100.9)	100.4 (98.2–101.4)	0.756		
Glucose (%)	Group A Control	99.7 (90.5–105.0)	103.2 (92.3–105.9)	0.722	0.566	0.363
	Group B Wine	101.1 (97–109.9)	105.1 (96.2–108.8)	0.422		
	Group C Ethanol	97.6 (95.5–105.2)	100.4 (96–101.5)	0.392		
Triglycerides (%)	Group A Control	92.8 (76.7–117.0)	101.3 (64.8–119.4)	0.554	0.805	0.312
	Group B Wine	100.9 (85.3–120.4)	103.6 (87.9–121.7)	0.923		
	Group C Ethanol	110.5 (77.9–114.7)	105.9 (91.2–123.5)	0.705		
Total Cholesterol (%)	Group A Control	94.4 (87.3–104.8)	103.7 (87.0–105.3)	0.241	0.461	0.969
	Group B Wine	100.5 (92.2–107.6)	98.9 (95.5–111.0)	0.955		
	Group C Ethanol	96.8 (89.9–110.8)	104.2 (94.4–105.8)	0.099		
HDL-c (%)	Group A Control	99.0 (92.5–102.6)	98.7 (96.4–105.4)	0.798	0.400	0.976
	Group B Wine	101.0 (96.1–113.1)	98.5 (94.9–114.7)	0.499		
	Group C Ethanol	102.0 (93.0–104.1)	100.3 (96.4–100.3)	0.477		
LDL-c (%)	Group A Control	91.9 (79.5–106.7)	103.0 (87.1–110.5)	0.165	0.468	0.979
	Group B Wine	101.2 (90.1–108.9)	100.0 (91.8–116.3)	0.654		
	Group C Ethanol	92.1 (87.3–111.5)	101.4 (94.9–109.5)	0.127		
Uric acid (%)	Group A Control	100.7 (93.1–107.1)	100.0 (86.4–107.2)	0.920	0.734	0.520
	Group B Wine	101.4 (93.6–110.1)	99.1 (95.0–115.1)	0.988		
	Group C Ethanol	100.3 (97.7–106.4)	102.2 (92.1–105.8)	0.112		
Insulin (%)	Group A Control	106.4 (98.7–125.2)	114.9 (84.2–117.9)	0.108	0.314	0.183
	Group B Wine	96.1 (88.7–110.2)	107.9 (86.2–122.9)	0.418		
	Group C Ethanol	100.7 (72.2–113.2)	93.4 (70.9–93.4) *	0.030		
HOMA IR (%)	Group A Control	104.9 (91.9–118.9)	100.8 (86.5–121.9)	0.873	0.885	0.333
	Group B Wine	97.7 (89.3–121.6)	104.8 (82.1–139.4)	0.580		
	Group C Ethanol	110.3 (66.8–118.7)	93.1 (66.7–103.3)	0.086		
SGOT-AST (%)	Group A Control	100.0 (84.0–105.8)	101.2 (94.1–106.9)	0.377	0.902	0.929
	Group B Wine	98.2 (82.7–110.0)	100.0 (90.4–110.8)	0.841		
	Group C Ethanol	97.9 (93.5–105.5)	102.0 (89.3–107.5)	0.285		
SGPT-ALT (%)	Group A Control	100.0 (83.4–106.9)	108.0 (88.2–110.5)	0.343	0.521	0.697
	Group B Wine	91.6 (78.1–109.2)	100.0 (84.1–114.9)	0.208		
	Group C Ethanol	101.6 (90.8–108.2)	84.2 (72.3–114.2)	0.890		
γGT (%)	Group A Control	100.0 (81.0–105.1)	100.7 (84.3–102.3)	1.000	0.520	0.696
	Group B Wine	100.0 (91.7–111.3)	97.9 (91.4–112.7)	0.764		
	Group C Ethanol	102.5 (83.4–117.6)	104.5 (81.9–131.1)	0.962		

Data are presented as median (25th–75th quartiles). Friedman’s two-way ANOVA by ranks was used for the estimation of the time effect in each intervention group (P_time_). Time-related pairwise comparisons were performed using a Wilcoxon signed Ranks Test. * indicates significant difference compared to baseline. Kruskal–Wallis test was used for the comparison of the three different intervention groups. BMI; body mass index, HDL; high-density lipoprotein, LDL; low-density lipoprotein, HOMA IR; homeostatic model assessment for insulin resistance, γ-GT; gamma glutamyltransferase, SGOT/AST; serum glutamic oxaloacetic transaminase/aspartate aminotransferase, SGPT/ALT; serum glutamic pyruvic transaminase/alanine aminotransferase. ^†^ *p* value from Friedman or Kruskal–Wallis.

**Table 4:** % Change of baseline values in oxidative stress biomarkers.

	Group	% Change 4 Weeks	% Change 8 Weeks	P_time_^†^	P_trial_^†^ 4 Weeks	P_trial_^†^ 8 Weeks
Oxidized guanine species (%)	Group A Control	92.6 (74.6–132.8) ^a^	90.6 (79.9–104.2) ^a^	0.142	0.004	<0.000
	Group B Wine	75.6 (52.2–100.0) *^,a^	85.0 (64.4–106.9) *^,a^	0.012		
	Group C Ethanol	108.6 (97.5–156.4) ^b^	131.1 (98.2–101.4) *^,b^	0.002		
Protein carbonyls (%)	Group A Control	97.4 (94.6–107.2) ^a^	95.4 (88.5–103.7) ^a,b^	0.195	<0.000	0.002
	Group B Wine	89.5 (72.6–94.0) *^,b^	84.4 (75.8–97.2) *^,a^	0.001		
	Group C Ethanol	111.2 (91.5–126.0) ^a^	118.1 (88.8–203.7) *^,b^	0.341		
Serum GPx activity (%)	Group A Control	98.6 (96.6–103.1)	98.3 (94.1–104.9)	0.125	0.290	0.893
	Group B Wine	97.6 (92.1–102.2) *	100.6 (90.9–105.2)	0.432		
	Group CEthanol	100.4 (95.4–103.3)	93.4 (93.1–108.4)	0.705		
LRP GPx activity (%)	Group A Control	95.3 (85.9–99.9)	95.6 (84.3–111.3)	0.094	0.219	0.550
	Group B Wine	102.1 (84.4–144.6) ^#^	86.4 (58.2–110.2)	0.142		
	Group C Ethanol	93.1 (65.6–105.6)	97.4 (91.5–102.7)	0.387		
SOD activity (%)	Group A Control	106.4 (82.1–122.6)	97.5 (85.3–125.0)	0.850	0.339	0.356
	Group B Wine	130.2 (78.4–161.5)	85.0 (56.7–137.6)	0.385		
	Group C Ethanol	103.0 (60.7–123.4)	105.5 (75.6–125.4)	0.705		
		4 weeks	8 weeks	P_time_^^^	P_trial_^^^	P_time*trial_^^^
TBARS (%)	Group A Control	98.9 ± 25.8	97.9 ± 24.7	0.197	0.775	0.898
	Group B Wine	96.9 ± 21.8	91.9 ± 17.2			
	Group C Ethanol	99.6 ± 31.4	92.3 ± 24.3			

For skewed variables, data are presented as the median (25th–75th quartiles). Friedman’s 2-way ANOVA by ranks was used for the estimation of the time effect in each intervention group (P_time_). Time-related pairwise comparisons were performed using Wilcoxon signed Ranks Test. * indicates significant difference compared to baseline, ^#^ compared to 8 weeks. Kruskal–Wallis test was used for the comparison of the three different intervention groups (P_trial_). Different letters indicate statistical significance. For normally distributed variables, data are presented as means ± SD. Repeated measures ANOVA was used for the comparisons. TBARS; thiobarbituric acid substances, GPx; glutathione peroxidase, LRP; leukocyte-rich plasma, SOD; superoxide dismutase. ^†^ *p* value from Friedman or Kruskal–Wallis. ^^^ p trend from RMANOVA.
